# Frequency of Self-Reported Concussion Amongst Professional and Semi-Professional Footballers in Ireland During the 2014 Season: a Cross-Sectional Study

**DOI:** 10.1186/s40798-017-0118-8

**Published:** 2018-01-08

**Authors:** Nicola Coffey, Martin Lawless, Seamus Kelly, Conor Buggy

**Affiliations:** 1 UCD Centre for Safety and Health at Work, School of Public Health, Physiotherapy and Sports Science, University College Dublin, Dublin, Ireland; 2 UCD Centre for Sports Studies, School of Public Health, Physiotherapy and Sports Science, University College Dublin, Dublin, Ireland

**Keywords:** Concussion, Occupational risk, Occupational injury, Safety awareness, Professional football

## Abstract

**Background:**

This paper examines the occupational risk of concussion amongst professional and semi-professional footballers in Ireland during the 2014 League of Ireland season. As part of a broader nationally representative study examining occupational safety and health (OSH) awareness amongst professional footballers, this empirical quantitative study, utilising a convenience sample is the first and largest investigation of the frequency of, and attitudes towards, concussion and concussion reporting amongst Irish senior professional and semi-professional footballers.

**Methods:**

A census survey using an anonymous questionnaire was provided to available League of Ireland clubs between March and May 2015. Permission to access players was provided by the Professional Footballers Association of Ireland. This convenience sample was determined by club availability in relation to match fixtures. Participation by the footballers was voluntary. At the time, there were 250 professional and semi-professional players within the League available to participate.

**Results:**

A total of 149 footballers participated in the study. Sixty percent of the participants were employed on a semi-professional basis and the majority of all participants were aged between 18 and 30. 15.7% of the participants reported having received a concussion in the 2014 season with semi-professional players having a noticeably higher (though not significant) reporting rate. Analysis indicated that there was a significant association between playing position and concussion reporting with defenders having the greater odds of reporting a concussion than other playing positions. Professional and semi-professional footballers have a relatively equal risk of receiving a concussion.

**Conclusion:**

This research is the first major investigation of the self-reported frequency of, and attitudes towards, concussion amongst Irish senior professional and semi-professional footballers. The results have important implications for coaches, clinicians, parents, players and national governing bodies. Further research is needed to ascertain whether professional footballers perceive concussion as an occupational risk, and whether they appreciate that accepting such risks can have long-term implications for health.

## Key points

- There is no association between self-reported concussion with professional status.
- Defenders are more likely to self-report a concussion.
- Sixteen percent of footballers that reported concussive symptoms were officially diagnosed as having received a concussion.

## Background

In 2013, the American National Football League agreed to pay $765 million in compensation to more than 4500 former professional players due to concussions and head injuries sustained during their careers [1]. In 2015, this compensation was increased to $914 million and extended to 5000 former professionals [2]. In 2002, a verdict of death by industrial disease was returned following an inquest into the death of former England professional footballer Jeff Astle. This inquest identified a type of dementia that was consistent with heading the ball. Consequently, considerable media attention drew the general public’s attention to the potential dangers and long-term consequences of sports-related concussion. Head trauma and concussion are everyday occupational risks for professional and semi-professional athletes competing in contact sports. However, these risks are not limited to elite athletes. In a quantitative epidemiological study of the general population of New Zealand [3], it was found that 21% of all traumatic brain injuries were sustained during sports-related activities. Despite increased public awareness and growth in concussion-related research, a number of concerns and important gaps in our knowledge still exist.

The 5th International Conference on Concussion in Sport (Berlin 2016), defined sports-related concussion as “a traumatic brain injury induced by biomechanical forces” [4]. In the USA [5], between 1.6 and 3.8 million athletes participating in a sporting activity suffer a concussion. However, the true figure is likely to be much higher, owing to many injuries not being recognised or reported [6]. While this has been a recognised player safety and health issue for over a decade [7], little progress has been made in reporting practice. Moreover, while many instances of sports-related concussion may be deemed clinically mild, there is evidence emerging that an athlete’s ability to fully recover from a concussion may be impaired and that the effects of repeated concussions are more severe than previously understood [5].

In recent years, there has been a growth in research examining concussion in football [8], with national football associations [9], national football leagues [10] and in countries such as the Netherlands [11] and Italy [12]. Studies of high-school [13], university-level [14] and professional [15, 16] football players exist. However, the significance of parameters such as player age and playing position remains unclear [17]. Moreover, despite a growth in studies examining concussion-reporting rates and practices with professional rugby players in Ireland [18, 19] studies examining the occupational risks associated with concussion amongst Irish semi-professional and professional footballers are lacking. Finally, research examining personal safety awareness and the frequency of concussion reporting amongst professional athletes has been limited. In response, the purpose of this study was to investigate the safety awareness and concussion-reporting frequencies of a cohort of Irish professional and semi-professional footballers.

## Methods

### Population

The study cohort consisted of professional and semi-professional football players playing in the top two divisions of the League of Ireland, which is run by the Football Association of Ireland (FAI, the governing body for football in the Republic of Ireland). Players may be classified by a number of factors such as age, gender and level of play (youth, university, amateur and professional) [17]. Semi-professional football players typically get paid and sign a semi-professional contract but may have other employment. Amateur players, regardless of age or playing level, do not get paid. At the commencement of the study in March 2015, it was indicated that there was a total of 250 professional (100) and semi-professional (150) footballers registered with the Professional Footballers Association of Ireland (PFAI). All were male and ranged in age from 18 to 39. These footballers had received educational information regarding concussion during the interval between the 2013 and 2014 seasons. In order to assess occupational safety awareness in League of Ireland footballers, certain inclusion and exclusion criteria were taken into consideration when evaluating the eligibility of potential applicants. The inclusion criteria were that each participant had to be registered with a League of Ireland football club, in receipt of remuneration for playing for the club, to be a member of the PFAI, and to have played professionally or semi-professionally for at least one season prior to 2014. Exclusion criteria were that players under 18 years of age, players that had not played professionally prior to the 2014 season and players on loan from non-League of Ireland clubs were excluded from the study.

### Survey

A census survey utilising an anonymous questionnaire was considered as the preferred method for obtaining safety awareness and self-reported concussion information. Permission was sought from and granted by the FAI to access their cohort of players to participate in the survey. Ethical exemption was granted by the University College Dublin Human Research Ethics Committee in advance of the study (no vulnerable groups, sensitive topics or conflicts of interest were identified). The survey was in the format of a questionnaire that was adapted from four appropriate and validated questionnaires identified: the Occupational Safety Climate Questionnaire [20]; the Organisational Practices Questionnaire [21]; the Questionnaire Measuring Perception of Workplace Safety [22]; and the Questionnaire on Occurrence of Concussion and Knowledge in Italian Soccer [12]. The latter questionnaire was based on the McCrea et al.*’s* Standardised Assessment of Concussion (SAC) [23]. For this study, the following questions were asked, based on that survey:

- During the 2014 season, did you experience any of the concussive symptoms, or have a concussion playing football, even if you did not tell anyone?
- How many concussions can you recall you received during the 2014 season?
- If you received a concussion, how many days did you have the symptoms, did you report your injury to anyone and how many days did you sit out due to the injury? [This question was asked for each concussion recalled].
- If you reported a concussion, to whom did you report it?
- If you did not report a concussion, what was your reason for not reporting it?

Due to population access permission restrictions, no questions were asked regarding; actions or inaction by management or support staff, attitudes towards management or support staff, attitudes towards or actions of peers in relation to safety and concussion or recall of specific concussion symptoms. Players were provided with a definition of concussion and a list of concussive symptoms for reference in answering the questions regarding their reporting. The questionnaire was piloted in advance on eight former professional footballers. Players could choose not to participate as it was entirely voluntary and anonymous and in no way could any individual be subsequently identified from the data gathered. By completing the questionnaire, participants implied consent for their responses to be included in the analysis and subsequent reporting.

### Data collection

Originally, the questionnaire was distributed via the online tool, Survey Monkey, for ease of completion. A link to the survey was emailed to the PFAI administration staff, along with the participant information sheet, in mid-March 2015. The link and participant information sheet were then forwarded to all members of the association. However, after 3 weeks, the response rate was low, rendering the study infeasible. Consequently, and in recognition of the importance of minimising the period of recall, it was determined that face-to-face distribution of the survey in hardcopy to the participants before training sessions would improve the response rate. Due to time constraints (all data was required to be gathered by mid-June 2015), a convenience sample of willing and available league clubs was utilised to expedite the completion of the data collection process. Hardcopy questionnaires were distributed between April and June 2015 (5 months after the conclusion of the 2014 season) at times convenient for the participating clubs. This method of convenience sampling while the most efficient to gain access to as many players as possible in a limited time, is acknowledged as a limitation to this research as discussed later. The questionnaires were filled out prior to training sessions. An additional question was added to hardcopy questionnaires, asking participants to confirm that they had not already completed the questionnaire online. In total, 68 footballers participated online while 81 footballers participated via the hardcopy questionnaire, giving a total of 149 participants and a response rate of 60%.

### Statistical analysis

Descriptive statistics were used to determine the population’s demographic profile, playing positions, frequency of concussions reported and reasons for non-reporting. Statistical analysis was conducted using SPSS with Pearson Chi-Square with Yates Correction and Fishers Exact utilised for testing. Odds ratios (OR) were also reported. Pearson Chi-square tests were used to compare the frequencies of self-reported concussion between groups for categorical variables such as age group (i.e. 18-29 years and 30–39 years), level of play (i.e. professional and semi-professional), and playing position (i.e. defenders, midfielders, and attackers). Pearson chi-square was utilised for initial significance testing and this was followed by or checking using a Yates correction and Fishers Exact tests as necessary. Fisher’s exact test was used when the chi-square criterion of a minimum cell number of five was not achievable. Any results of *p* < 0.05 at the 95%CI were deemed significant. Some comments were added based on the merit of the 90%CI results in some cases.

## Results

Just over one third of the participants of the study (*n* = 54) were professional footballers, with the remaining participants designated as semi-professional (*n* = 95). The majority of both professional footballers and semi-professional footballers were aged (years = a) between 18a and 29a; *n* = 35 (64.8%) and *n* = 80 (84.3%) respectively. 34 participants were aged between 30a and 39a, the majority of which were professional footballers; *n* = 19 (55.8%). Significantly more players from the 18a to 29a age category participated in the study as indicated by a Pearson chi-sq test (*p* = 0.007, OR 2.9 (1.3–6.4)) reinforced by a Fishers exact test (0.013) and the Yates correction (0.012) all to the 95% CI.

15.7% of the total population reported having officially received a verified concussion in the 2014 season (N.B. 127 out of 149 participants answered this question). There was no significant difference between professional and semi-professional players regarding self-reporting a concussion. This was confirmed via a Pearson chi-sq (*p* = 0.43, OR 1.5 (0.5–4.5)) and supported by a Fishers exact (0.6) and Yates correction (0.6).

Table 1 provides a breakdown of self-reported concussion based on playing position. Some players designated themselves as playing in multiple positions. Amongst the defenders, there was a significant difference between professional and semi-professional players as regards the frequency of self-reported concussion. Table 1 shows significance within the defender group reporting concussion with a Pearson chi-square result (*p* = 0.04, OR 5.0 (1.0-36.9)). The Fishers exact was less significant for this group (0.09) and the Yates correction reinforces this (0.09) although when one looks at the analysis at the 90%CI both Fishers (0.05) and Yates (0.05) show significance at that level indicating that there is some merit in the result. None of the other playing positions show any material significance and this is backed up by both the Fishers and Yates analysis.

**Table 1:** Self-reported concussion associated with playing position and professional status

Position and Status	Yes		No		Total		OR	*P* value
	*n*	(%)	*n*	(%)	*n*	(%)		
Goalkeeper								
Pro	0	(0)	7	(100)	7	(100)		
Semi-pro	0	(0)	9	(100)	9	(100)	1.3 (0.2–73.2)	0.9
Defender								
Pro	2	(11)	16	(89)	18	(100)		
Semi-pro	10	(39)	16	(61)	26	(100)	*5.0 (1.0–36.9)*	*0.045*
Defender/midfielder								
Pro	0	(0)	2	(100)	2	(100)		
Semi-pro	0	(0)	2	(100)	2	(100)	1.0 (0.01–80.0)	0.99
Midfielder								
Pro	2	(12)	15	(88)	17	(100)		
Semi-pro	2	(4)	22	(96)	24	(100)	2.8 (0.2–90.1)	0.38
Attacker								
Pro	2	(50)	2	(50)	4	(100)		
Semi-pro	3	(18)	14	(82)	17	(100)	4.2 (0.5–47.6)	0.17

Further statistical analysis indicated that when considering the defenders as a single group (*n* = 44, 35%) versus the rest of the players (*n* = 83; 65%) irrespective of professional status, there was a greater likelihood of self-reported concussion. 12 defenders self-reported concussion as opposed to 8 other players (Pearson chi-square *p* = 0.009, OR 3.5 (1.3–9.7)). This was further reinforced with the Fishers exact test (0.02) and the Yates correction (0.02) at the 95%CI.

Players can report concussive symptoms to a wide range of individuals within their support network, including teammates and club employees. Table 2 indicates to whom players reported their symptoms. There were no significant differences between professional or semi-professional players in this regard.

**Table 2:** Individuals to whom concussive symptoms were reported

	Physio		Coach		Teammate		Doctor		Parent		Total	
Status	*n*	(%)	*n*	(%)	*n*	(%)	*n*	(%)	*n*	(%)	*n*	(%)
Pro	7	(44)	1	(6)	2	(13)	5	(31)	1	(6)	*16*	*(24)*
Semi-pro	22	(42)	11	(21)	2	(4)	13	(25)	4	(8)	*52*	*(76)*
*Total*	*29*	*(43)*	*12*	*(18)*	*4*	*(6)*	*18*	*(27)*	*5*	*(7)*	*68*	*(100)*

Few players admitted to suffering concussive symptoms but failing to report them. As Table 3 shows, no significant differences were reported between professional and semi-professional players in this regard, however the semi-pro players had the higher level of non-reporting concussive symptoms.

**Table 3:** Reason for not reporting concussive symptoms

Reason	Pro		Semi-Pro		Total	
	*n*	(%)	*n*	(%)	*n*	(%)
Did not want to let teammates down.	0	(0)	2	(100)	*2*	*(25)*
Did not want to miss a game.	0	(0)	0	(0)	*0*	*(0)*
Unaware it was concussion.	0	(0)	2	(100)	*2*	*(25)*
Not serious enough.	1	(25)	3	(75)	*4*	*(50)*
*Total*	*1*	*(13)*	*7*	*(87)*	*8*	*(100)*

Of those whose concussion was verified after they reported concussive symptoms, only 60% (12 out of 20) reported being prevented from playing or training as a consequence. Table 4 indicates that there was no significant difference between professional and semi-professional players in this regard.

**Table 4:** Days benched or omitted from training/playing after reporting

Duration (days)	Pro		Semi-pro		Total	
	*n*	(%)	*n*	(%)	*n*	(%)
0–5	2	(25)	6	(75)	8	*(67)*
6–10	2	(50)	2	(50)	4	*(33)*
11+	0	(0)	0	(0)	0	*(0)*
*Total*	*4*	*(33)*	*8*	*(67)*	*12*	*(100)*

## Discussion

Of the 127 players who responded to the questions regarding concussion within this broader study, 68 reported concussive symptoms to individuals within their support network. Of these 68 players, 20 (15.7%) were officially diagnosed with concussion. This figure is considerably higher (> 10%) than that of a similar representative study conducted with Italian footballers, which found that 5% of the population had suffered a concussion in the previous season [12]. Globally, footballers’ risk of concussion is considered to be substantial, accounting for a quarter of all injuries amongst elite male players [24, 25]. Factors impacting football players’ self-reporting, or under-reporting, of concussion include the importance of a particular match, an eagerness to return to play for big games, the possibility of being prevented from playing and the availability of quality substitutes [15]. However, the higher levels of recognition of concussive symptoms and of self-reported concussion for League of Ireland players in the 2014 season could be attributed to a number of factors. While concussion research was previously focussed on the validation of concussion assessment tools, more recent studies have emphasised the need for improved concussion education for stakeholders in football [26]. There has also been significant mainstream media interest in concussion in sport over the last few years. Consequently, it is argued that players are now more aware of concussion (types, symptoms, sources, diagnosis, treatment etc.) in general [27], and of the need to report it to relevant personnel in particular [28]—and indeed, are more likely to do so [29].

This higher levels of reporting are of interest because, while increased concussion incidence and reporting by players may impact team selection in the short term, the potential recurrence of concussion can impact career opportunities and trajectory in the long term. A career in professional football is characterised by chronic insecurity and uncertainty [30]. For professional footballers, missing a game through injury can have considerable short-term consequences in terms of getting back into the team as well as more significant long-term implications in terms of securing of a professional contract [30]. Furthermore, the threat of deselection is more significant for professional footballers and players may hide injuries, not impacting performance, from medical staff and managers [30]. The moderately higher reporting levels of semi-professional footballers in this study could be due, in part, to this cohort possessing alternative occupations, employment opportunities and sources of income. In contrast to the findings of recent research in professional football in England [15], Irish semi-professional footballers may be less risk averse, may connect their personal actions with the risk and may be more aware of the long-term impact of concussion. Thus, while concussion education has improved considerably recently, changing the culture of underreporting will require ongoing efforts over many years [29]. As we know, concussion injuries may have an associated stigma, viewed as a ‘less serious injury than a leg break’ (15:201). In this regard, there is a need for further player and manager education linking the short-term decision to ignore concussion symptoms with the long-term potential risks.

This study’s results indicate that certain playing positions have a greater likelihood of receiving a concussion than others. These results are similar to those of studies carried out with professional rugby league players, in which it was noted that the ball carrier (attacker) was found to be at greater risk of sustaining a concussion than the tacklers (defender), probably because rugby league is a full contact sport [31]. An Irish study carried out with rugby union players came to a similar conclusion, with a higher proportion of concussions being sustained by attacking players; however, interestingly, the severity of concussions was greater for defending players [17]. Regarding the age ranges of both the professional and semi-professional players in this study, the professional players had an older age range and their experiences with concussion over their careers may be influencing the reporting practices of the younger members of their teams.

Despite semi-professional footballers accounting for over two thirds of the concussions reported in the study, professional status does not appear to have an association with concussion. While professional sport is a unique ‘work’ environment, legislation to protect workers is still applicable, depending on the jurisdiction. When considering concussion as an occupational injury for professional athletes, comparison with other occupational sectors can give an insight into how injuries are perceived in professional sport. Across the EU, concussion accounted for 17% of all workplace injuries in 2013 [32]. In contrast, the Irish Central Statistics Office (CSO) noted that concussion, grouped with several other occupational injuries (including amputation, internal injury, burn, scald or frostbite) made up only 7% of all workplace injuries in 2013 [33]. While it is not possible to obtain an exact figure for the number of workplace concussions, it is worth noting that the total number of injuries in this group nationally was 1300. Consequently, 20 concussions could be considered high for professional and semi-professional footballers as one defined occupational group. Given that concussion in professional sport can be considered an occupational risk, it is imperative that professional athletes’ awareness of this risk should be evaluated more rigorously [34].

There are a number of issues to bear in mind when interpreting the results of the current study. One methodological limitation is the sample size. In this regard, it must be noted that gaining access to professional football players and clubs normally presents difficulties for researchers, as professional football clubs are often quite wary of social science researchers [15] and when exploring sensitive issues such as concussion in particular. Moreover, it is often difficult to secure the cooperation of professional leagues, professional football associations and governing bodies. Consequently, concussion-related research in football utilising professional football players, though growing [11, 15, 16], is limited. A second limitation concerns recall error. Asking athletes to recall symptoms from the previous year when they may not have been able to recognise them or were unwilling to report them can lead to bias, which has implications for the internal validity of the study [35, 36]. However, this study occurred immediately after the preceding season when the players had returned to pre-season training, thus minimising recall duration [35]. Despite its limitations, retrospective recall does ‘allow an athlete the opportunity to reveal symptoms that may not have been identified prospectively’ (14: 337). However, mitigating measures to enhance validity and minimise self-report limitations such as miscomprehension, measurement error and conscious bias [34, 35] were adopted. For instance, participants were provided with clear instructions and an overview of the rationale of the study. It was also assumed that participants were ‘honest in their responses without a societal response bias’ (15: 202). Situational limitations, such as confidentiality [34], were addressed by excluding coaches, managers and support staff. This, coupled with the anonymous nature of the survey, allowed the players to participate and answer truthfully without fear of and potential negative repercussions [34]. This could be a factor in the higher reporting levels evidenced in this study.

## Conclusions

This research is the first and largest investigation of the self-reported frequency of, and attitudes towards, concussion amongst Irish senior professional and semi-professional footballers. This study has indicated that professional status does not have a significant bearing on the likelihood of recognising concussive symptoms or of reporting a concussion. Further research is needed to ascertain whether professional footballers perceive concussion as an occupational risk, and whether they appreciate that accepting such risks can have long-term implications for health (i.e. that cumulative sporting injuries can potentially lead to debilitating health conditions). As a questionnaire may not be an effective tool for exploring attitudes towards concussion, it has been argued that understanding player’ views on self-reporting concussion would require qualitative research [15].
